# Scientific evidence supports aerosol transmission of SARS-COV-2

**DOI:** 10.1186/s13756-020-00868-6

**Published:** 2020-12-18

**Authors:** C. Raina MacIntyre, Michelle R. Ananda-Rajah

**Affiliations:** 1 Biosecurity Research Program, Kirby Institute, UNSW Medicine, Sydney, Australia; 2 Department of General Medicine, Alfred Health, Melbourne, Australia

We question the evidence cited by Conly et al. [1] to justify recommending masks for routine care of COVID-19 patients. As evidence, the authors cite the R0 and include several references that are not primary research, and only two primary studies. One of these is a study of hospital contamination which found evidence of surface contamination within a hospital but was negative for air samples [2]. This same study is used as evidence supporting contact and fomite transmission, but other studies which did find virus in air samples were disregarded [3–6] Ong et al. found evidence of virus on hospital air vents, but this is disregarded, and the meaning of finding viral RNA in air samples is questioned by Conly et al. This represents shifting goalposts for proving airborne transmission of SARS-COV-2, which was initially denied altogether, then changed to questioning the infectious potential of air in which viral RNA is found, to later questioning the infectious dose required in air, after viable virus was demonstrated in the air [6]. In fact, viable SARS-COV-2 has been found in the air in hospital rooms in the absence of aerosol generating procedures [6].

The other cited evidence is lack of transmission on an aircraft while the index case was symptomatic [7]. The majority of transmission occurs in the 48 h prior to symptom onset and in the first 6 h of symptoms, with a declining infectious function thereafter, so if the cited case was already symptomatic, he was likely less infectious while flying [8]. Further, the lack of transmission on board this aircraft could equally be used to “disprove” droplet transmission, given other passengers would have been seated within 2 m of the patient, so this is not credible evidence. In fact, there have been other airplane outbreaks, as well as outbreaks on buses that support aerosol transmission [9, 10]. Long range faecal aerosol transmission of SARS-COV-2 in an apartment block has also been documented [11].

The authors incorrectly cite the R0 of SARS-COV-2 as evidence to support droplet transmission. The R0 is not, and has never been a criterion for defining the mode of transmission. R0 is a function of the pathogen, the host and the environment, and varies for any given pathogen by factors such as population density and environment. As such, it is not a scientific measure of transmission mode, and cannot be used selectively to support droplet transmission of SARS-COV-2. Accepted estimates for the R0 of SARS-COV-2 are between 2 and 4 [12], but as high as 6 in New York State and Wuhan [13, 14]. Given that over 80% of cases are mild, and there is substantial asymptomatic infection, the official case counts upon which the R0 is calculated are likely a vast under-estimate, and R0 is likely higher.

Tuberculosis, which is accepted as airborne, has a R0 range which lower than that of SARS-COV-2 [15]. Influenza, too, has been shown repeatedly to be capable of aerosol transmission and found in the air hours after an infectious patient has left the room [16, 17], despite having a lower R0 range than SARS-COV-2 [18]. Pertussis is as infectious as measles, with a R0 range up to 18, but is classified as droplet transmitted [19], again highlighting that R0 is not a valid measure of mode of transmission. One reason is the infectious dose, which differs for different airborne pathogens, and explains why tuberculosis has a much lower R0 than measles—the difference is explained by a much lower infectious dose of measles. This complexity also illustrates why R0 cannot be used to determine transmission mode. Further, the infectious dose of SARS-COV-2 is still unknown, and this uncertainty in itself should warrant a precautionary approach.

The authors correctly cite measles and tuberculosis as airborne infections, but neither has ever been isolated as viable pathogens from air samples [20, 21], whereas SARS CoV2 has [6]. Airborne transmission has been subject to a much higher burden of proof than droplet or contact transmission of SARS-COV-2, and also to a higher evidence standard compared to other pathogens. This is the opposite of a precautionary approach in the face of uncertainty.

The cost of persevering with an argument based on selective evidence is the safety and lives of health workers, who are being denied airborne precautions by health authorities all over the world. Many guidelines still advocate the surgical mask which is not actually designed or approved for respiratory protection. The best available data on beta coronaviruses show superior protection offered by N95 respirators compared to surgical masks [22]. Finally, transmission mode is only one consideration in making guidelines for PPE. Other criteria include work health and safety obligations, the presence of scientific uncertainty, immunity, the availability of drugs and vaccines for the disease and the seriousness of the infection [23]. Getting it wrong for a serious infection such as COVID-19 matters much more than getting it wrong for a trivial infection. The same dogmatic arguments about droplet versus airborne precautions occurred in Toronto in 2003 during SARS, with a failure to provide airborne precautions for health workers, a subsequent outbreak and health worker deaths. The SARS Commission in Toronto recommended the use of the precautionary principle to protect health workers during a serious emerging infectious threat [24]. The case for the acceptance of the science around airborne transmission coupled with precautionary control measures has never been more compelling than during the COVID-19 pandemic.
